# A Novel On‐Chip Method for Differential Extraction of Sperm in Forensic Cases

**DOI:** 10.1002/advs.201800121

**Published:** 2018-06-15

**Authors:** Fatih Inci, Mehmet O. Ozen, Yeseren Saylan, Morteza Miansari, Duygu Cimen, Raghu Dhara, Thiruppathiraja Chinnasamy, Mehmet Yuksekkaya, Chiara Filippini, Deepan Kishore Kumar, Semih Calamak, Yusuf Yesil, Naside Gozde Durmus, George Duncan, Leonard Klevan, Utkan Demirci

**Affiliations:** ^1^ Bio‐Acoustic MEMS in Medicine (BAMM) Laboratory Canary Center at Stanford for Cancer Early Detection Department of Radiology Stanford School of Medicine Stanford University Palo Alto CA 94304 USA; ^2^ Department of Medicine Brigham and Women's Hospital Harvard Medical School Boston MA 02115 USA; ^3^ Department of Biochemistry Stanford University Stanford Genome Technology Center Palo Alto CA 94304 USA; ^4^ Crime Laboratory Broward County Sheriff's Office Fort Lauderdale FL 33301 USA; ^5^ DxNow Inc. Gaithersburg MD 20879 USA; ^6^ Department of Electrical Engineering (by courtesy) Stanford University Stanford CA 94305 USA

**Keywords:** bioinspired materials, differential extraction, DNA casework backlog, forensic cases, microfluidics

## Abstract

One out of every six American women has been the victim of a sexual assault in their lifetime. However, the DNA casework backlog continues to increase outpacing the nation's capacity since DNA evidence processing in sexual assault casework remains a bottleneck due to laborious and time‐consuming differential extraction of victim's and perpetrator's cells. Additionally, a significant amount (60–90%) of male DNA evidence may be lost with existing procedures. Here, a microfluidic method is developed that selectively captures sperm using a unique oligosaccharide sequence (Sialyl‐Lewis^X^), a major carbohydrate ligand for sperm‐egg binding. This method is validated with forensic mock samples dating back to 2003, resulting in 70–92% sperm capture efficiency and a 60–92% reduction in epithelial fraction. Captured sperm are then lysed on‐chip and sperm DNA is isolated. This method reduces assay‐time from 8 h to 80 min, providing an inexpensive alternative to current differential extraction techniques, accelerating identification of suspects and advancing public safety.

## 1 Introduction

The failure to test and analyze evidence connected to sexual assault in a timely manner constitutes a growing problem for victims, public safety, and the criminal justice system. The Rape, Abuse & Incest National Network has reported that a sexual assault occurs in every 98 s in the United States alone, with the majority of victims being under the age of 30.^1^, ^2^, ^3^ An investigative report in 2015 identified over 70 000 sexual assault kits from over 1000 police departments (≈6% of the police departments in the USA) that were not tested for DNA evidence.^4^ Therefore, the demand for DNA testing is increasing. Expanded awareness of the power of forensic technology to help in solving crimes creates new needs for scientific advances in the field.^5^, ^6^ Among these advances, microfluidic technologies have considerable impact by combining high‐throughput processing and efficient isolation of cells and biological entities from complex heterogeneous biological matrices.^7^, ^8^, ^9^

**Figure 1:**
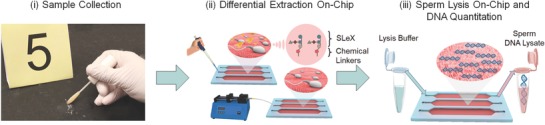
Workflow of on‐chip differential extraction. i) In practice, samples are collected using a swab or cotton gauze in a forensic scene, where a mixture of semen and epithelial cells are majorly present on the victim's body and/or garments at the crime scene. ii) After collection, samples are simply introduced into the device using single‐step pipetting and incubated for an hour at room temperature. The channels are then washed and sperm cells are specifically captured, while epithelial cells are removed due to their larger size and lack of an adhesion molecule on the channel surface. iii) The captured sperm are treated with a lysis buffer on‐chip, and sperm DNA is collected into a tube for potential forensic downstream genomic analyses.

In practice, processing of evidence from sexual assault kits generally requires separation of the victim's cells from the perpetrator's cells. This process involves time‐consuming, labor‐intensive steps of selective cell lysis, centrifugation, and separation into female and male cell fractions (i.e., differential extraction) which can take up to 8 h, contributing significantly to the backlog problem. However, it has been reported that this cell separation process results in losses of 60–90% of the male DNA.^10^, ^11^, ^12^, ^13^ Although there have been multiple attempts for alternative methods to differentially extract sperm using acoustic trapping,^14^ antibody‐based capture,^15^ laser microdissection,^16^, ^17^, ^18^ nuclease‐based approaches,^19^ and magnetic bead‐based separation,^20^, ^21^ these methods have not been broadly available in practical applications due to the complexity and low separation yield for sperm. As a result, they are not widely in use in the community. Particularly, the antibody‐based extraction methods have difficulties to work with aged samples due to the changes in the antigen specificity of sperm over time. Hence, this challenge makes them less capable to capture sperm, which decreases to ≈17% after 10 days, limiting their utility and applicability for forensic samples.^21^ To address these unmet challenges, we have developed a microfluidic method integrated with a bioinspired oligosaccharide sequence for selective isolation, differential extraction, and quantitation of sperm from the forensic evidence of heterogeneous cellular content in sexual assault kits (**Figure** **1**). Here, we present a method that i) differentially isolates sperm and lyses them on‐chip, and extracts sperm DNA for downstream genetic analyses; ii) reduces the differential extraction time from 8 h to 80 min; iii) minimizes the need for manual labor; iv) increases capture efficiency of immuno‐based separation of sperm assays from ≈17%^21^ to 70–92%; and v) keeps this high efficiency for samples older than 15 years, representing a crucial direction to reduce the evidence backlog.

## 2 Results and Discussion

### 2.1 Molecular Docking Study

A recent study identified an oligosaccharide (i.e., Sialyl‐Lewis^X^ (SLeX: [NeuAcα2‐3Galß1‐4(Fucα1‐3)GlcNAc])) as a unique molecule that sperm uses to bind to the egg.^22^, ^23^ Although this study did not define exact mechanisms of binding, it created a new direction to bind sperm selectively to surfaces, circumventing the degradation problem that is inherent to antibodies that focus on the sperm surface for immuno‐separation purposes.

**Figure 2:**
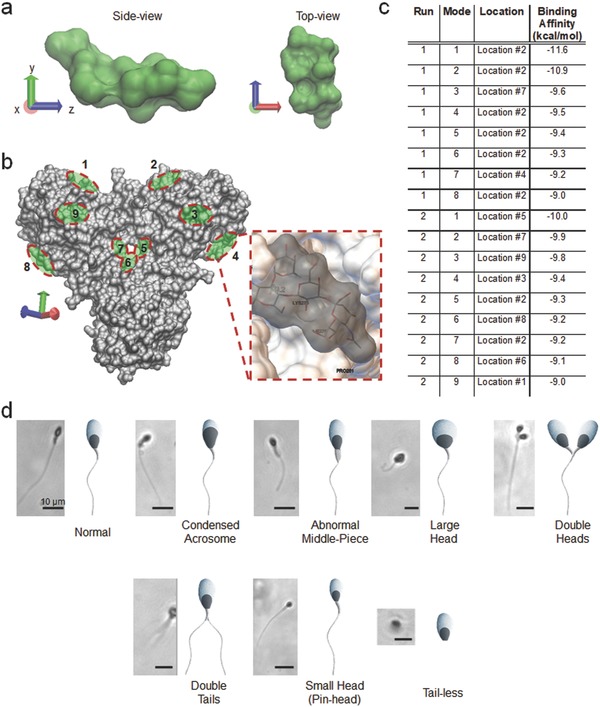
Evaluation of SLeX binding kinetics and binding locations on sperm head. a) SLeX structure was extracted from a protein complex defined in the Protein Data Bank (PDB ID: 3PVD) and visualized in silico. Computational analysis revealed the molecular surface of the SLeX agent for sperm binding using VMD's built‐in SURF tool. b) β1–4 galactosyltransferase 1 (B4GALT1) was extracted from human M340H‐beta‐1,4‐galactosyltransferase‐1 (M340H‐B4GAL‐T1, PDB ID: 4EE3) and visualized in silico. This enzyme‐receptor on the sperm plasma membrane plays a key role in sperm‐egg binding. B4GAL‐T1‐SLeX interactions were then computed using AutoDock Vina, and the analyses revealed at least nine unique locations for seventeen potential binding modes for SLeX binding to B4GALT1. c) At these docking sites, strong binding was observed with the affinity energies ranging from −9.0 to −11.6 kcal mol^−1^. d) We further observed that SLeX molecules capture sperm cells with different morphologies (i.e., normal, condensed acrosome, abnormal middle‐piece, large head, double heads, double tails, small head, and tail‐less) on‐chip. These experimental findings confirmed our results observed in silico, indicating that SLeX targets sperm head and its binding is independent of distinct sperm morphologies. Scale bars (black lines) represent 10 µm.

In the experiments, we utilized this bio‐inspired material, i.e., SLeX, which is located on the extracellular matrix (i.e., zona pellucida (ZP)) of oocyte, as a capture agent (Figure S1, Supporting Information). This oligosaccharide sequence has been reported as a major contributing element for human sperm‐oocyte binding.^22^, ^23^ There are also components on the sperm membrane reported as docking units, including the β1–4 galactosyltransferase 1 (B4GAL‐T1) peripheral protein, which plays a crucial role in human sperm‐oocyte binding.^24^, ^25^, ^26^, ^27^, ^28^, ^29^ To understand the dynamics of SLeX binding to sperm surface, we used B4GAL‐T1 as a model docking/binding unit on the sperm membrane and computed a molecular docking simulation to discover the locations and energetics of binding (**Figure** **2**). In this process, as shown in Figure 2a, we first extracted molecular structure of SLeX in silico from a protein complex defined in the Protein Data Bank (PDB ID: 3PVD).^30^ We then extracted B4GAL‐T1 from human M340H‐beta‐1,4‐galactosyltransferase‐1 (M340H‐B4GAL‐T1, PDB ID: 4EE3) (Figure S2a, Supporting Information).^31^ The results of the docking simulations of molecular surfaces of SLeX and B4GAL‐T1 were computed and visualized using AutoDock Vina and Visual Molecular Dynamics (VMD). The docking analysis revealed seventeen potential binding modes with at least nine different locations on the B4GAL‐T1 surface for SLeX binding (Figure 2b–d and Figure S2b–e, Supporting Information). This study revealed strong binding modes with affinity energies ranging from −9.0 to −11.6 kcal mol^−1^ (Figure 2c). We observed a binding hot‐spot at the Location #2, where eight of the seventeen SLeX molecules were bound (Figure S2c and Video S1, Supporting Information). Experimentally, we also confirmed that SLeX decorated microfluidic surfaces was able to capture sperm with various morphologies, including normal, condensed acrosome, abnormal middle‐piece, large head, double heads, double tails, small head (pin‐head), and tail‐less (Figure 2d). Given that SLeX targets the sperm head, binding and capture of sperm was independent of sperm morphology. Specifically, sperm without a tail were also captured with SLeX agent primarily interacting with the sperm head.

### 2.2 Evaluating Surface Characteristics and Sperm Capture Efficiency in Microchannels

To efficiently capture sperm in microchannels, we integrated SLeX with a microfluidic technology. Briefly, we designed microchannels that consist of three layers: i) poly(methyl methacrylate) (PMMA) for formation of inlets and outlets, ii) double‐sided adhesive (DSA) for the formation of microchannels and the assembly of one PMMA and one glass layer, and iii) glass coverslip surface (Figure S3, Supporting Information). Layer‐by‐layer, physical and chemical modifications are applied to the glass surface to immobilize SLeX (Figure S4, Supporting Information).

**Figure 3:**
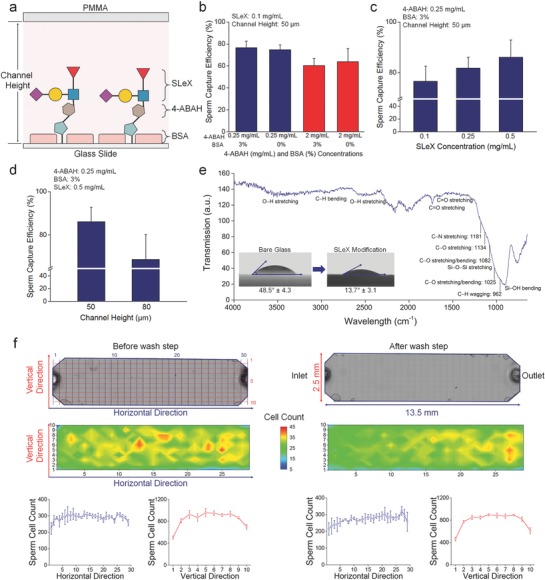
Evaluation of surface chemistry and microfluidic chip parameters for sperm capture. a) Glass surfaces were decorated with SLeX agent using a layer‐by‐layer surface chemistry approach. Capture efficiency was evaluated by varying three parameters: i) concentration of mediator molecule (i.e., 4‐Aminobenzoic acid hydrazide: 4‐ABAH) and bovine serum albumin (BSA), ii) SLeX concentration, and iii) channel height. b) Various 4‐ABAH (0.25 mg mL^−1^ and 2 mg mL^−1^) and BSA concentrations (0% and 3%) were examined, and sperm capture efficiency was calculated at each concentration. In these experiments, 50 µm high microchannels were modified with a fixed SLeX concentration (0.1 mg mL^−1^). Here, 0.25 mg mL^−1^ of 4‐ABAH provided higher capture efficiency than 2 mg mL^−1^ of 4‐ABAH but it was not statistically different (*n* = 3–4, *p* > 0.05). Further, this might be due to potential steric hindrance for SLeX immobilization to the surface. Further, BSA blocking did not significantly affect the sperm capture efficiency (*n* = 3–4, *p* > 0.05) in these experimental sets. c) Different SLeX concentrations ranging from 0.1 to 0.5 mg mL^−1^ were used to evaluate sperm capture. The 50 µm high microchannels were modified with the optimized 4‐ABAH (0.25 mg mL^−1^) and BSA (3%) concentrations. Here, 0.5 mg mL^−1^ of SLeX concentration provided higher capture efficiency compared to the other groups. Further, the capture efficiency in 0.5 mg mL^−1^ of SLeX was not statistically different than the other SLeX concentrations (*n* = 4, *p* > 0.05). d) Two channel heights (50 µm and 80 µm) were evaluated in terms of sperm capture efficiency. The microchannels were decorated with the optimized 4‐ABAH (0.25 mg mL^−1^), BSA (3%), and SLeX (0.5 mg mL^−1^) concentrations. We observed that 50 µm high channel heights resulted in higher capture efficiency than an 80 µm high channel. e) Surface functionalization and SLeX binding on the modified channels were confirmed by Fourier Transform Infrared‐Attenuated Total Reflectance (FTIR‐ATR) measurements. At the fingerprint region of SLeX (900 cm^−1^ to 1280 cm^−1^), we observed C—H wagging at 962 cm^−1^, C—O stretching/bending at 1025 and 1082 cm^−1^, C—O stretching at 1134 cm^−1^, and C—N stretching at 1181 cm^−1^. Due to characteristic absorption region of glass between ≈682 cm^−1^ and ≈1200 cm^−1^ (e.g., Si—OH bending and Si—O—Si stretching), the signal intensities of peaks in this region reduced. We also observed absorption peaks at 3500, 2850–2950, 2500–3000, 1733, and 1630–1690 cm^−1^ caused by O—H stretching, C—H bending, O—H stretching, C=O (ester) stretching, and C=O (amide) stretching vibrations of SLeX molecule, respectively. By performing contact angle measurements, we evaluated hydrophilicity properties after surface modification (inset figure). The contact angle value of the bare glass surface altered from 48.5° ± 4.3 to 13.7° ± 3.1 after SLeX modification. According to the FTIR‐ATR and contact angle measurements, SLeX molecule was successfully immobilized to the microchannel surface. f) Spatial distribution of cell capture was analyzed on‐chip by imaging the entire microchannel surface through a tiling function of the microscope with an automated *x*–*y* stage. Sperm counts before and after the washing step were plotted through horizontal and vertical directions. Before the washing step, a homogenous cell distribution was observed in a horizontal direction, whereas sperm cell count increased in the middle of the channels on the vertical axis. The cell count was altered in the horizontal direction after the washing step and most of the sperm close to the inlet washed away from the channel surface. On the other hand, the distribution trend at the vertical axis did not change after the washing step. For statistical analysis, we used one‐way ANOVA with Tukey's post hoc test for multiple comparisons with the statistical significance threshold set at 0.05 (*p* < 0.05). Data is represented with average value ± standard deviation (*n* = 3–4).

Capture efficiency was assessed by varying three main parameters: i) concentration of mediator agent (i.e., 4‐aminobenzoic acid hydrazide: 4‐ABAH) and evaluation of bovine serum albumin (BSA) blocking, ii) SLeX concentration, and iii) channel height (**Figure** **3**a). We first examined the effect of 4‐ABAH concentrations (0.25 and 2 mg mL^−1^) on sperm capture efficiency, keeping the SLeX concentration (0.1 mg mL^−1^) and microchannel height (50 µm) constant. We observed higher capture efficiencies at 0.25 mg mL^−1^ of 4‐ABAH concentration but it was not statistically different (*n* = 3–4, *p* > 0.05) (Figure 3b). Further, this may point to a potential steric hindrance in higher mediator concentrations for SLeX immobilization. As reported in the literature, more densely packed layers revealed lower surface activity.^32^, ^33^ This effect also indicated the link between surface coverage, immobilization of molecules and capture activities, and therefore, a lower density of immobilization process on the surface provided a higher binding and sensitivity.^32^, ^34^ Since 4‐ABAH has a role to immobilize SLeX molecules in the next step, much lower concentrations of 4‐ABAH will potentially not provide more binding points for binding of SLeX agent to the surface. Therefore, we determined to use 0.25 mg mL^−1^ of 4‐ABAH in the experiments. In addition, we utilized BSA as a blocking agent, which has ideally dual roles: i) blocking agent blocks nonmodified spots in the microchannel to minimize nonspecific binding of the other cells/biological entities (e.g., epithelial cells); and ii) it does not significantly affect sperm capture while minimizing the nonspecific binding. In the experiments, BSA blocking did not significantly change sperm capture efficiency (*n* = 3–4, *p* > 0.05). To minimize nonspecific binding, we also utilized BSA in the specificity experiments to capture sperm from complex heterogeneous cell population including epithelial cells. This experimental set achieved a 76.5 ± 6.0% of capture efficiency when 0.25 mg mL^−1^ of 4‐ABAH and 3% of BSA were applied with the other constant parameters of SLeX and channel height. Next, we evaluated the effect of SLeX concentrations varying from 0.1 to 0.5 mg mL^−1^ over sperm capture efficiency, keeping the microchannel height (50 µm), 4‐ABAH (0.25 mg mL^−1^) concentration and BSA (3%) constant (Figure 3c). We observed that the increase in SLeX concentration enhanced sperm capture efficiency, and the highest SLeX concentration (0.5 mg mL^−1^) resulted in 86.1 ± 6.8% of capture efficiency by generating more binding sites for sperm capture. Further, this increase in capture efficiency was not statistically different than the other SLeX concentrations (*n* = 4, *p* > 0.05). Finally, we evaluated the effect of microchannel height on sperm capture efficiency when we kept the aforementioned concentrations (4‐ABAH: 0.25 mg mL^−1^ and SLeX: 0.5 mg mL^−1^). Given that increased surface interactions are vital for cell capture, we observed higher capture efficiency with 50 µm high channel design compared to 80 µm high channel design (Figure 3d). Overall, the highest sperm capture efficiency was achieved using i) 0.25 mg mL^−1^ of 4‐ABAH and 3% BSA, ii) 0.5 mg mL^−1^ of SLeX, and iii) 50 µm high microchannel. We applied these parameters to the following experimental designs to capture sperm. In the experiments, we also observed that sperm cells were tightly captured in microchannels. Although sperm were trying to move, they were also stuck to the channel surface due to high capture capacity of SLeX material (Video S2, Supporting Information).

To confirm surface functionalization and SLeX binding, we performed Fourier Transform Infrared‐Attenuated Total Reflectance (FTIR‐ATR) measurements on the modified microchannels (Figure 3e). As the fingerprint region of SLeX was designated between 900 and 1280 cm^−1^,^35^ we first analyzed this range and observed C—H wagging at 962 cm^−1^, C—O stretching/bending at 1025 and 1082 cm^−1^, C—O stretching at 1134 cm^−1^, and C—N stretching at 1181 cm^−1^. These absorbance spectra values appear to be shifted from unbound SLeX molecule,^35^ which might be caused by the generation of chemical bonding during immobilization process. Since glass has intense characteristic spectrum between ≈682 cm^−1^ and ≈1200 cm^−1^ (e.g., Si—OH bending and Si—O—Si stretching),^36^, ^37^, ^38^ the signal intensities of peaks in this region reduced. Further, the absorption peak around 3500 cm^−1^ represented O—H stretching vibrations due to high number of free O—H groups on SLeX molecule. The intensity band appearing around 2850–2950, 2500–3000, 1733, and 1630–1690 cm^−1^ were caused by C—H bending, O—H stretching, C=O (ester) stretching, and C=O (amide) stretching vibrations of SLeX molecule, respectively.^39^ We further characterized hydrophilicity of microchannel surface after the modification (Figure 3e‐inset). The contact angle value of the bare glass surface reduced from 48.5° ± 4.3 to 13.7° ± 3.1, indicating that SLeX immobilization with layer‐by‐layer surface chemistry generated more hydrophilic surface. These two different characterization methods confirmed that SLeX molecule was successfully immobilized to the microchannel surface.

### 2.3 Evaluating Distribution of Sperm Capture in Microchannels

We assessed the spatial distribution of sperm on‐chip by counting sperm before and after phosphate buffered saline (PBS) washing steps. In this experiment, we applied high and low sperm counts into the channels. During the imaging studies, the entire channel was divided into 30 columns (horizontal direction) by 10 rows (vertical direction). First, we evaluated ≈8000 sperm per channel (high sperm count) (Figure 3f). Before the washing step, we observed homogenous distribution of sperm in a horizontal direction, whereas higher cell numbers were counted in the middle of the channel while scanning the vertical‐axis. After the washing step, the cell count decreased in the first 5–10 lanes close to the inlet in the horizontal direction. On the other hand, the vertical distribution did not change after the washing step. In the second experimental set, we applied a lower sperm count (≈300 sperm per channel) (Figure S5, Supporting Information). Before the washing step, we observed nearly homogenous cell distribution in a horizontal direction. Through the vertical axis, we observed the same trend as with higher sperm count experiments, and the sperm cell count was higher in the middle of channel. After the washing step, the sperm count close to the inlet was altered in a horizontal direction, which was similarly observed in higher sperm count experiments. After washing, the vertical axis also had a similar distribution trend, as observed before the washing step.

### 2.4 Benchmarking Nonspecific Cell Binding (Control)

**Figure 4:**
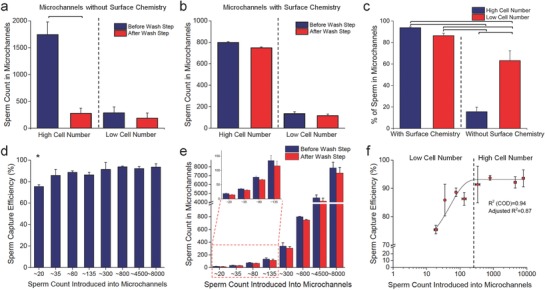
Evaluation of nonspecific sperm cell binding (control) and limit of detection. a) The microchannels without surface chemistry were used as a control set. Nonspecific sperm cell binding was assessed with high (750–1800 sperm per channel) and low (100–300 sperm per channel) cell numbers. Only a limited number of sperm (275 ± 96 cells) remained in the channels when we applied 1742 ± 239 cells into the microchannels. Sperm samples with a high cell number were significantly removed from the channel surfaces in the absence of surface chemistry (*n* = 4, *p* < 0.05). In addition, some sperm (186 ± 97 cells) remained when we introduced 285 ± 111 cells to the microchannels (*n* = 4, *p* > 0.05). These results demonstrated that the bare glass surface itself has ≈200 nonspecific binding points over the sperm count range. b) We also evaluated the detection capability of microchannels modified with surface chemistry. Most sperm (748 ± 9 cells) were captured when we applied 798 ± 9 cells into the microchannels (*n* = 3, *p* > 0.05). In low cell count experiments, we observed that 116 ± 17 sperm were captured on‐chip when we introduced 134 ± 19 cells to the microchannels (*n* = 3, *p* > 0.05). As demonstrated in the plot, the microchannels modified with surface chemistry efficiently captured sperm in both high and low cell numbers with ≈94% and ≈86% efficiency, respectively. c) Further, cell numbers were converted into percentage of sperm remaining in microchannels after washing step. Higher ratios of sperm remained on the surface chemistry applied channels than that of control surfaces without surface chemistry (*n* = 3–4, *p* < 0.05). We also observed that ≈200 bindings were mainly due to sperm‐glass surface interactions in control surfaces (*n* = 4, *p* < 0.05). In these experiments, we introduced 15 µL of samples with high and low sperm counts into the channels. d,e) We evaluated the limit of detection parameter for the microchannels by applying multiple cell concentrations varying from ≈20 to ≈8000 cells per channel. The microchannels captured down to ≈20 sperm cells per channel with a capture efficiency of 75.4 ± 1.5% (*n* = 3, *p* < 0.05), and the capture efficiency increased up to 93.6 ± 3.0% at higher cell counts (up to ≈8000 cells per channel), indicating that the microchannels were able to handle a broad range of cell numbers and the capture capability of chips was independent of high cell numbers introduced into the microchannels. f) Limit of detection parameter was further analyzed through a nonlinear fitting function. The curve had a linearity of 0.94 and 0.87 for *R* ^2^ (Coefficient of determination: COD) and adjusted *R* ^2^, respectively. For statistical analysis, we used one‐way ANOVA with Tukey's post hoc test for multiple comparisons with the statistical significance threshold set at 0.05 (*p* < 0.05). Horizontal brackets and asterics demonstrate statistically significant differences between groups. Data is represented with average value ± standard deviation (*n* = 3–4).

In control experiments, we did not decorate the channels with surface chemistry, and the glass surface was only cleaned with EtOH before being assembled (**Figure** **4**). We then introduced 15 µL of samples with high and low sperm counts into the channels. High cell counts were defined as being between 750 and 1800 sperm per channel, whereas the low cell count was around 100–300 sperm per channel. In high cell count experiments, only a limited number of sperm remained (275 ± 96 cells) in the control surfaces without surface chemistry when we applied 1742 ± 239 cells to the channels (Figure 4a). As a result, sperm samples with high cell counts were significantly removed from the channel surfaces in the absence of surface chemistry (*n* = 4, *p* < 0.05). In low cell count experiments, some sperm (186 ± 97 cells) remained when we introduced 285 ± 111 cells to microchannels without surface chemistry (*n* = 4, *p* > 0.05) (Figure 4a). In control channels for both high and low cell count experiments, we observed that the bare glass surface itself had ≈200 nonspecific cell adherence points over all sperm count ranges introduced into the channel.

After that, we further evaluated sperm counts in the channels modified with surface chemistry (Figure 4b). In high cell count experiments, most sperm (748 ± 9 cells) were captured when we applied 798 ± 9 cells into microchannels (*n* = 3, *p* > 0.05). In low cell count experiments, 116 ± 17 sperm were captured in the channels when we introduced 134 ± 19 cells to the microchannels (*n* = 3, *p* > 0.05). Comparing the data between surface chemistry applied channels and control surfaces (no surface chemistry) in high cell count experiments, a high ratio of sperm (≈94%) was captured on the surface chemistry decorated channels, whereas cells were significantly removed in control channels and only ≈16% of sperm remained in the control channels (Figure 4b). We further analyzed the data and converted cell numbers into percentage of sperm remaining in microchannels after washing step (Figure 4c). In both high and low cell count experiments, greater ratios of sperm remained on the surface chemistry applied channels than that of control surfaces, indicating that surface chemistry (SLeX coating) has significant effect on sperm capture efficiency for both low and high sperm concentrations applied to the channels (*n* = 3–4, *p* < 0.05). Additionally, we observed the nonspecific cell adherence effect on the control surfaces, and ≈200 bindings were mainly due to sperm‐glass surface interactions (*n* = 4, *p* < 0.05). These ≈200 nonspecific binding points were consistent for both low and high concentrations applied to the channels, indicating the highest limit of nonspecific binding for the current channel size, geometry, and surface chemistry. Overall, the microchannels modified with surface chemistry efficiently captured sperm with a range of 86–94% in both high and low cell count experiments.

### 2.5 Evaluating Limit of Detection (LOD)

We assessed this parameter by applying multiple cell counts (≈20 to ≈8000 sperm per channel) into the channels and calculating capture efficiency at each cell concentration (Figure 4d–f). As a result, the channels captured down to ≈20 sperm per channel with a capture efficiency of 75.4 ± 1.5%, and capture efficiency increased up to 93.6 ± 3.0% at higher cell counts (at ≈8000 sperm per channel) (Figure 4d,e). Therefore, the microchannels were able to handle a broad range of cell numbers and the capture capability of microfluidic chips was independent of high cell counts introduced into the channels. Statistical assessments demonstrated that capture efficiency derived from ≈20 sperm per channel experiment was lower than the other cell concentration groups (*n* = 3–9, *p* < 0.05), and also, there was no statistical difference between ≈35 and ≈8000 sperm per channel (*n* = 3–9, *p* > 0.05) (Figure 4d,e). Since surface chemistry and channel parameters were all same, there might be two reasons: i) the binding sites of SLeX in the microchannels might be saturated while applying samples with higher sperm counts and this might have helped the capturing performance of SLeX; and ii) washing step might be more effective over capture efficiency in samples with lower sperm counts since sampling size varies by ≈20 – ≈8000 sperm per channel. This could potentially contribute to changes in capture efficiency parameter due to different effective ratio of sperm removed from the surface after washing step. Further, in the experiments, we observed a nonlinear trend with 0.94 and 0.87 for *R* ^2^ (Coefficient of determination: COD) and adjusted *R* ^2^, respectively. The curve was also examined in two regions: i) low cell count (≈20 to ≈300 sperm per channel), and ii) high cell count (≥ 300 sperm per channel). Samples lower than 300 sperm per channel range provided a capture efficiency between 75.4% and 86.3%, whereas the capture efficiency for above 300 sperm per channel reached up to 93.6 ± 3.0% (Figure 4f).

### 2.6 Evaluating Specificity of Sperm Capture in Microchannels

**Figure 5:**
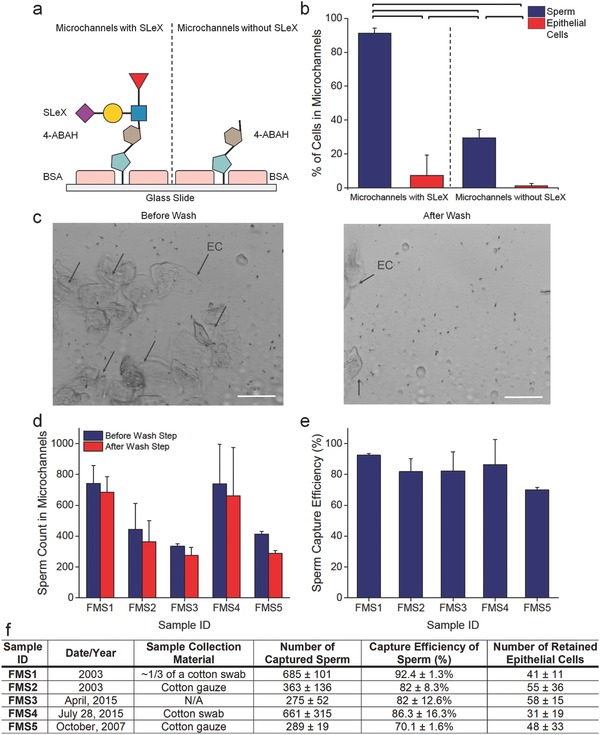
Specificity experiments and validation of microfluidic chips with forensic mock samples. a) Specificity of SLeX was tested with a heterogeneous cell population consisting of a male's sperm and buccal epithelial cells collected from a female's inner cheeks. Two sets of microfluidic chips were prepared: i) all surface chemistry steps including SLeX and ii) all surface modifications without SLeX. b) SLeX‐modified surfaces provided 91.1 ± 3.1% of capture efficiency, whereas sperm cells drastically washed away from the surfaces without SLeX (*n* = 5, *p* < 0.05). In addition, SLeX provided high specificity to capture sperm (≈91%) compared to epithelial cells (≈7% and ≈1%) in both experimental sets (*n* = 5, *p* < 0.05). There was no significant binding of epithelial cells observed in the microchannels with SLeX and without SLeX (*n* = 5, *p* > 0.05). c) Microphotography was performed before and after the washing steps on microchannels with SLeX. Black arrows represent epithelial cells (ECs) in the microchannels. Scale bars represent 50 µm. d) Simulated forensic samples (noncasework samples) were obtained from the Broward Sheriff's Office Forensic Laboratory. Five different mock samples were introduced into the microchannels modified with SLeX, and the numbers of sperm were then counted before and after the wash steps. We observed various numbers of sperm ranging from ≈300 to ≈745 cells, and most of the sperm cells were captured in the microchannels. e) Mock samples provided high capture efficiencies, spanning from ≈70% to ≈92%. f) The mock samples were collected, using either cotton swab or cotton gauze, on different dates and they consisted of different cell content and concentrations. The details were presented in the table. Data was represented with average value ± standard deviation (*n* = 3). For statistical analysis, we used one‐way ANOVA with Tukey's post hoc test for multiple comparisons with the statistical significance threshold set at 0.05 (*p* < 0.05). Horizontal brackets demonstrate statistically significant differences between groups. Data is represented with average value ± standard deviation (*n* = 5).

Vaginal samples in sexual assault kits typically contain vaginal epithelial cells from the victim and sperm cells from the perpetrator. To evaluate specificity performance of microfluidic chips, we designed two experimental sets: i) microchannels surfaces decorated with SLeX molecules, and ii) microchannels surfaces modified up to the 4‐ABAH binding step (non‐SLeX). In both experimental sets, we worked with a heterogeneous cell population including sperm and buccal epithelial cells. Thus, we evaluated whether SLeX is crucial in specific capture of sperm from mixed cell populations (**Figure** **5**). In these experiments, the entire microchannel was scanned to count sperm and epithelial cells before and after washing steps (Figure S6, Supporting Information). On SLeX‐modified surfaces, the percentage of captured sperm cells (≈91%) was statistically greater than nonspecifically bound epithelial cells (≈7%) (*n* = 5, *p* < 0.05). Considering the necessity of SLeX to capture sperm, we observed a drastic decrease in the percentage of captured sperm on non‐SLeX surfaces (*n* = 5, *p* < 0.05). No statistical difference was observed in the percentage of remaining epithelial cells in both non‐SLeX and SLeX‐coated channels (*n* = 5, *p* > 0.05). Overall, in these experiments, we obtained two critical outcomes: i) SLeX‐modified surfaces specifically captured sperm and a vast majority of epithelial cells (≈93%) were removed after a single wash step; and ii) SLeX played a pivotal role in capturing and isolating sperm from a heterogeneous cell population (Figure 5b,c and Figure S6, Supporting Information).

### 2.7 Validating Microfluidic Chip Performance with Forensic Mock Samples

Forensic mock samples were collected from the Broward Sheriff's Office Forensic Laboratory. In validation studies, samples were sent to Stanford University under the approved IRB protocol. The collected samples were noncasework/mock samples, including epithelial cells and sperm. According to the guidelines of Broward Sheriff's Office Forensic Laboratory, five mock samples from 2003 to 2015 were collected with either cotton swab or cotton gauze, and directly introduced through SLeX‐decorated channels with three replicates (Figure 5d). Sperm cells were counted before and after washing steps. In the forensic mock samples, we observed a various number of sperm and epithelial cells. Sperm after washing step was counted in the microchannels, and high capture efficiencies ranging from ≈70% to ≈92% were observed for aged mock samples (Figure 5e,f). Additionally, as reported in the literature, cotton content interferes with capture performance of assays,^21^ and we observed similar hindrance when a large cotton swab was used. For instance, in the FMS2 and FMS5, the capture efficiency decreased to ≈82% and ≈70%, respectively. Whether a full size of cotton swab or just a portion of cotton swab was used, the capture efficiency ranged from 86% to 92% (FMS1 and FMS4). We also counted the retained epithelial cells in the channels and observed a significantly lower number of epithelial cells compared to the captured sperm count (*n* = 3, *p* < 0.05) (Figure S7, Supporting Information). As demonstrated in the spiking experiments, we also confirmed that our microchannels were able to specifically capture sperm from a heterogeneous cell population, and device performance was not significantly changed while capturing sperm from aged forensic mock samples.

### 2.8 Sperm Lysis On‐Chip and DNA Quantification

Captured sperm in microchannels were first treated with tris(2‐carboxyethyl)phosphine (TCEP) in Triton X‐100 to lyse cells on‐chip. The collected lysate solution was then processed through Proteinase K and spin column protocols, as described in the Materials and Methods section. After these protocols, the DNA concentration of each sample was measured and demonstrated in **Table** **1**. Since each sperm cell includes ≈3 pg of DNA material, the captured cell number was then converted into an expected DNA concentration of each sample. We counted different number of sperm ranging from 3160 to 7731 in the microchannels. After lysis step, we quantified DNA amount of collected samples within a range from 79 to 188 pg µL^−1^ (Table 1). According to all these results, we achieved sperm lysis on‐chip and confirmed high DNA recovery with efficiency ratios between ≈52.8% and ≈88.6%, demonstrating the applicability of our platform for potential forensic downstream analyses.

**Table 1:** Efficiency of sperm lysis on‐chip and quantification of lysed sperm DNA

Sample ID	Sperm count on‐chip	Expected DNA concentration [pg µL^−1^]	Qubit result[pg µL^−1^]	Efficiency (%)
S1	7731	≈289.9	188	≈64.8%
S2	4990	≈149.7	79	≈52.8%
S3	5237	≈159.8	91	≈57%
S4	3160	≈94.8	84	≈88.6%

## 3 Conclusion

The differential extraction of sexual assault samples from sexual assault kits requires up to 8 h of skilled personnel to complete. Even while performing lengthy sample process steps, a significant amount (60–90%) of male DNA may be lost during existing procedures as reported in the literature.^10^, ^11^, ^12^, ^13^ Here, we present a next‐generation differential extraction technology that is, to the best of our knowledge, the most rapid, reliable, accurate, user‐friendly method available. Although there are previously antibody‐based capture approaches proposed for forensic samples, they suffer from loss of efficiency and specificity over time since proteins on the sperm membrane aged over a long‐term storage, as well as during drying process.^15^, ^20^, ^21^ As we have shown in this study, SLeX has multiple binding sites on the sperm surface, making it a unique element for aged forensic sperm samples, allowing our methods to achieve ≈5‐fold higher sperm capture efficiency. Our technology solves a significant problem that has failed to find a solution in the past for efficient differential extraction of sperm.

Here, we integrated microfluidics with a unique oligosaccharide unit (i.e., SLeX), a major binding ligand for egg and sperm interaction. By introducing bioinspired materials into a microfluidics realm, we have developed a powerful platform to selectively isolate sperm in heterogeneous matrices by performing only few steps (four sampling/washing and two incubation steps) to provide on‐chip sperm DNA lysate within 80 min. All sampling and extraction steps can be performed by existing forensic DNA laboratory equipment and techniques such as sample loading with a pipette and a single‐flow rate wash for controlling selective removal of unbound cells from microchannels. We validated this procedure with forensic mock samples shelved for over a decade, and we succeed to differentially capture sperm cells in channels with high capture efficiency (70–92%).

This method is still in development, and we expect the following improvements in the design and workflow. First, the current design of chips has up to 4 microchannels and process 5–15 µL of sample volume per channel, which is typical in a case sample. By integrating various designs of channel lateral dimensions and numbers, the platform can potentially handle larger sample volumes for high‐throughput DNA extraction. Second, as the incubation time for sperm capture takes 75% of total processing time, this assay time would potentially be further reduced by decreasing channel height. Third, although the current system uses a simple hand‐pipette and a syringe pump in the sampling and washing steps, the entire platform can potentially be automated by integrating an automatic pipetting system, as well as creating a closed‐box system that minimizes personnel integration and person‐to‐person variability. Also, automated preparation techniques using a robotic arm could considerably minimize potential batch‐to‐batch variations. Fourth, in this study, sexual assault kit samples were visualized using a standard laboratory microscope. The next generation of this platform can potentially be integrated with a portable imaging system,^40^ enabling easy access to the technology in remote locations for sexual assault evidence screening. Fifth, we decorated channels with SLeX monomer units in the current study. Higher concentrations of SLeX (more than 0.5 mg mL^−1^) can also be utilized to evaluate its effect over sperm capture efficiency. Ideally, a bioprinting strategy will further accelerate to increase the coverage rate of SLeX in the channels. Sixth, although the present platform utilizes affordable components such as plastic layers, polymers, and glass slides, the cost of goods used for the fabrication and surface chemistry can potentially be reduced further with mass production.

Overall, the presented microfluidic technology with a bio‐inspired oligosaccharide sequence addresses critical technical challenges in forensic rape cases, facilitating downstream genomic analyses, accelerating identification of suspects, and advancing public safety. In addition, the ability of our technology i) to differentially extract sperm from heterogeneous cell population, ii) lyse sperm on‐chip, and iii) extract sperm DNA within a short assay‐time can open up new avenues for forensic downstream analyses.

## 4 Experimental Section

*Materials*: (3‐Mercaptopropyl)trimethoxysilane (3‐MPS, 95%), aminobenzoic acid hydrazide (4‐ABAH, 95%), bovine serum albumin (BSA), dimethyl sulfoxide (DMSO), Triton X‐100, Proteinase K (recombinant, PCR Grade), and ethanol (EtOH, 200 proof) were purchased from Sigma‐Aldrich (St. Louis, MO). *N*‐γ‐maleimidobutyryl‐oxysuccinimide ester (GMBS), TCEP, and Qubit dsDNA High Sensitivity (HS) Assay were obtained from Thermo Fisher Scientific (Waltham, MA). PBS and SLeX were obtained from Fisher Scientific (Hampton, NH), Zymo Research (Irvine, CA), and EMD Millipore (Hayward, CA), respectively. QIAamp DNA Mini Kit was purchased from Qiagen (Valencia, CA).

*Molecular Docking Study*: A molecular docking simulation is employed to study the binding localization and energy of SLeX – M340H‐β‐1,4‐galactosyltransferase‐1 (M340H‐B4GAL‐T1 (B4GAL‐T1) interactions on sperm membrane. The structural coordinate data of SLeX and B4GAL‐T1 were extracted from the Protein Data Bank.^30^, ^31^ The molecular surfaces of SLeX and B4GAL‐T1, along with the results of the docking simulations, were computed and visualized using VMD.^41^ AutoDock Tools (ADT) 4.2 was utilized to configure the simulation input files.^42^ SLeX and B4GAL‐T1 were converted into the PDBQT file format. AutoDock Vina was then used for the molecular docking simulation,^43^ followed by another ADT run to assess ligand‐receptor hydrogen bonding and binding affinities. Binding affinities were reported as −kcal mol^−1^ for each interaction.

*Microchannel Fabrication*: The microfluidic chips consisted of three main components: i) a PMMA layer (3.2 mm of thickness), ii) a DSA film (50  and 80 µm of thickness), and iii) a glass cover slide (24 × 40 mm). Versa LASER (Universal Laser Systems Inc., Scottsdale, AZ) and CorelDRAW software (Ottawa, Ontario, Canada) were utilized to design and cut PMMA layers and DSA films. Inlets and outlets of the chips (0.65 mm in diameter, 26 mm apart) were milled into a PMMA layer, and the DSA film provided microfluidic channels. The microfluidic chips were then constructed by assembling these three components. Glass cover slides were used as a substrate material, where we performed surface chemistry for sperm capture.

*Surface Functionalization*: Glass cover slides were first cleaned with absolute EtOH (200 proof) via sonication for 15 min at room temperature. The slides were immediately dried under either N_2_ gas or filtered dry air, and then treated with oxygen plasma (ION3, Corona, CA) (100 mW, 1% oxygen) for 1.5 min to form radical groups. To generate thiol groups, the slides were placed into a 4% v/v solution of 3‐MPS in absolute EtOH and incubated for 30 min at room temperature. After the silanization step, the surfaces were rinsed with EtOH to remove unbound chemical residues and dried using either N_2_ gas or filtered dry air. After the microfluidic chip's three components were assembled, GMBS (10 × 10^−3^ m in DMSO:PBS (1:1)) was introduced into the microchannels to form succinimide groups by incubating for 45 min at room temperature. The microchannels were then washed with 1xPBS (40 µL, 2 times). 4‐ABAH reagent (0.25 and 2 mg mL^−1^ in 1:1 (v:v) ratio of DMSO:1xPBS) was utilized to form hydrazide groups for immobilization of SLeX molecules to the microchannels surface. After a washing step with 1xPBS (40 µL, 2 times), different concentrations of SLeX ranging from 0.1 to 0.5 mg mL^−1^ were prepared using the stock SLeX solution (1 mg mL^−1^) and applied to the microchannels. These surfaces were incubated overnight at +4 °C. The microchannels were then washed with 1xPBS (40 µL, 2 times) and the surface functionalization was accomplished with BSA (3% (w:v) in 1xPBS) incubation for an hour at room temperature to minimize/avoid nonspecific binding.

*FTIR‐ATR Spectroscopy*: After surface chemistry was applied to the microchannels, the PMMA and DSA layers were removed for FTIR‐ATR measurements. The SLeX functionalized mircochannel was then placed on the FTIR‐ATR instrument (Thermo Fisher Scientific, Nicolet iS10, Waltham, MA, USA) and total light reflection was recorded from 650 to 4000 cm^−1^ range with 2 cm^−1^ of resolution.

*Contact Angle Measurements*: KRÜSS Drop Shape Analyzer (DSA100, Hamburg, Germany) instrument was utilized for contact angle measurements. The contact angle values were recorded with sessile drop method by dropping 5 µL of ultrapure water and calculated as the average of the three different drops.

*Sampling and Counting*: For spiked sperm samples, sperm were purchased from California CryoBank under an Institutional Review Board (Stanford University IRB Number: 6208, and Protocol ID: 30538). Frozen sperm vials were briefly thawed in a water‐bath set at 37 °C, and the numbers of sperm in each sample were counted using a hemocytometer. Before sampling, sperm were incubated at room temperature for 1–3 d. For sampling, 5–15 µL of sample was applied into the microchannels to ensure the channels filled with the sample. Sperm samples were incubated for an hour while the imaging was being performed within the entire microchannel using a tiling function of the light microscope with a motorized *x*–*y* stage (Zeiss, Germany) (before the washing step). The microchannels were then washed with 1xPBS for 20 min using a syringe pump with a 5 µL min^−1^ flow rate to remove unbound cells. The captured cells within the microchannels were counted (after the washing step). A second imaging step was performed to count the number of captured sperm on‐chip. Sperm counts before and after the washing steps were manually calculated using these microscope images. The capture efficiency rate was defined as (Equation (1)

(1) $$\mathrm{Capture}\;\;\mathrm{Efficiency}\;\;\left(\%\right)\;\;=\;\;\frac{\mathrm{Sperm}\;\;\mathrm{count}\;\mathrm{after}\;\mathrm{washing}}{\mathrm{Sperm}\;\mathrm{count}\;\mathrm{before}\;\mathrm{washing}}\;\;\times \;\;100$$

In specificity experiments, buccal epithelial cells were collected from female individuals and were mixed with sperm samples. The specificity experiments also followed the same sampling procedure as described above.

*Forensic Mock Samples*: Simulated forensic samples were prepared by members of the Broward Sheriff's Office Crime Laboratory (not from casework evidence). Cuttings (cotton swab or cotton gauze) from these samples were eluted in 500 µL of 1 × PBS and placed in a 4 °C Thermomixer (Eppendorf, Germany) that was set at 1000 rpm for approximately an hour. The cuttings were removed and placed in spin baskets that were subsequently centrifuged for 5 min at 16 100 rcf/13 200 rpm to pellet the solids in the solution. Afterward, ≈300 µL of the 1 × PBS was removed without disturbing the pellet. The pellet was resuspended by pulse vortexing, and 5 µL of each sample was then placed on a slide, heat fixed, and dyed with a Christmas Tree stain as a confirmatory test before applying samples into the microchannels.^44^

*Sperm Lysis On‐Chip*: To lyse sperm cells and collect DNA on‐chip, TCEP was utilized as a lysis agent, and 20 µL of TCEP solution were introduced (20 µL of TCEP + 1980 µL of RNase free water + 20 µL of Triton X‐100 (100%), pH was adjusted to pH 2.5 with HCl) into the microchannel, and then, incubated for 15 min. An additional 80 µL of TCEP solution was applied into the channel, and the lysate was collected in an eppendorf tube.

After completion of cell lysis in all experimental sets, 40 µL of Proteinase K solution (1 µg mL^−1^) were added to each lysate tube and incubated for 4 h at 55 °C. During incubation, the tube inverted occasionally to disperse the sample. Followed by the incubation, 100 µL of Buffer AL and 100 µL of ethanol were added to the samples and mixed by vortexing. The samples were then run through gDNA extraction using a Qiagen spin column protocol.

*Qiagen Spin Column Protocol*: All samples were processed through the spin column procedure according to the manufacturer's protocol. The samples were applied to the QIAamp Mini spin column in a 2 mL collection tube without wetting the rim. The tubes were centrifuged at 6000 × *g* (8000 rpm) for 1 min. The QIAamp Mini spin column was placed in a clean 2 mL collection tube and the tube containing the filtrate was discarded. 500 µL of Buffer AW1 was then added without wetting the rim. The tubes were again centrifuged at 6000 × *g* (8000 rpm) for 1 min. After that, the QIAamp Mini spin column was placed in a clean 2 mL collection tube, and the collection tube containing the filtrate was discarded. 500 µL of Buffer AW2 was added without wetting the rim and centrifuged at full speed (20 000 × *g*; 14 000 rpm) for 3 min and the old collection tube with the filtrate was discarded. The tubes were again centrifuged at full speed for 1 min. The QIAamp Mini spin column was placed in a clean 1.5 mL microcentrifuge tube and the collection tube containing the filtrate was discarded. 50 µL of Buffer AE or distilled water was added. The tubes were then incubated at room temperature for 1 min and centrifuged at 6000 × *g* (8000 rpm) for 1 min. This step was repeated one more time. The final solution was ≈75–100 µL for each sample.

*Quantitation of Extracted DNA*: DNA concentration of each sample was quantified using the Qubit Fluorometer. The manufacturer's protocol for Qubit dsDNA High Sensitivity (HS) Assay (Thermo Fisher Scientific, Waltham MA) was followed. First, the Qubit working solution was prepared by diluting the Qubit dsDNA HS Reagent 1:200 in Qubit dsDNA HS Buffer. Then, two standards were prepared by adding 10 µL of standard solution into 190 µL of working solution. After that, sample solutions were prepared by adding 2 µL of each sample into 198 µL of working solution. All samples and standards were vortexed for 2–3 s without generating any bubbles and incubated for 2 min at room temperature. On the Qubit Fluorometer, a global curve was first generatued using two standards, and DNA concentrations of each sample were then measured. The data were represented as pg µL^−1^.

*Statistical Analysis*: One‐way analysis of variance (ANOVA) was employed with Tukey's posthoc test for multiple comparisons using GraphPad Prism (La Jolla, CA). The statistical significance threshold was set at 0.05 (*p* < 0.05).

## Conflict of Interest

Prof. Utkan Demirci (U.D.) is a founder of and has an equity interest in: i) DxNow Inc., a company that is developing microfluidic and imaging technologies for point of care diagnostic solutions, ii) Koek Biotech, a company that is developing microfluidic IVF technologies for clinical solutions, and iii) Levitas Inc., a company focusing on developing products for liquid biopsy. U.D.'s interests were viewed and managed in accordance with the conflict of interest policies.

## Supporting information

Supplementary

Supplementary

Supplementary
