# Supplementary material for: A Novel On‐Chip Method for Differential Extraction of Sperm in Forensic Cases
Source: Adv Sci (Weinh). 2018 Jun 15;5(9):1800121. doi: 10.1002/advs.201800121 (PMC6145299; doi:10.1002/advs.201800121)
Supplement: Supplementary file 1 — Supplementary [file ADVS-5-1800121-s002.pdf]

Copyright WILEY-VCH Verlag GmbH & Co. KGaA, 69469 Weinheim, Germany, 2018.

ADVANCED SCIENCE
Open Access

Supporting Information

for *Adv. Sci.,* DOI: 10.1002/advs.201800121

A Novel On-Chip Method for Differential Extraction of Sperm in Forensic Cases

Fatih Inci, Mehmet O. Ozen, Yeseren Saylan, Morteza Miansari, Duygu Cimen, Raghu Dhara, Thiruppathiraja Chinnasamy, Mehmet Yuksekkaya, Chiara Filippini, Deepan Kishore Kumar, Semih Calamak, Yusuf Yesil, Naside Gozde Durmus, George Duncan, Leonard Klevan, and Utkan Demirci**

Copyright WILEY-VCH Verlag GmbH & Co. KGaA, 69469 Weinheim, Germany, 2016.

Supporting Information

A novel on-chip method for differential extraction of sperm in forensic cases

*Fatih Inci, Mehmet O. Ozen, Yeseren Saylan, Morteza Miansari, Duygu Cimen, Raghu Dhara, Thiruppathiraja Chinnasamy, Mehmet Yuksekkaya, Chiara Filippini, Deepan Kishore Kumar, Semih Calamak, Yusuf Yesil, Naside Gozde Durmus, George Duncan, Leonard Klevan *, Utkan Demirci**

Dr. Fatih Inci, Dr. Mehmet O. Ozen, Dr. Yeseren Saylan, Dr. Morteza Miansari, Dr. Duygu Cimen, Raghu Dhara, Dr. Thiruppathiraja Chinnasamy, Semih Calamak, Yusuf Yesil, Prof. Utkan Demirci
Bio-Acoustic MEMS in Medicine (BAMM) Laboratory
Canary Center at Stanford for Cancer Early Detection
Department of Radiology, Stanford School of Medicine, Stanford University, Palo Alto, CA, 94304, USA

Dr. Mehmet Yuksekkaya, Chiara Filippini, Deepan Kishore Kumar
Department of Medicine, Brigham and Women's Hospital, Harvard Medical School, Boston, MA 02115, USA

Dr. Naside Gozde Durmus
Department of Biochemistry, Stanford University, Stanford Genome Technology Center, Palo Alto, CA 94304, USA

Dr. George Duncan
Crime Laboratory, Broward County Sheriff's Office, FL 33301, USA

Dr. Leonard Klevan
DxNow Inc., Gaithersburg, MD 20879, USA

Prof. Utkan Demirci
Department of Electrical Engineering (by courtesy), Stanford University, Stanford, CA 94305, USA

** co-corresponding authors: Utkan Demirci, Ph.D. (email: utkan@stanford.edu) and Leonard Klevan, PhD (email: lennyklevan2@gmail.com)*

Supplementary Figures

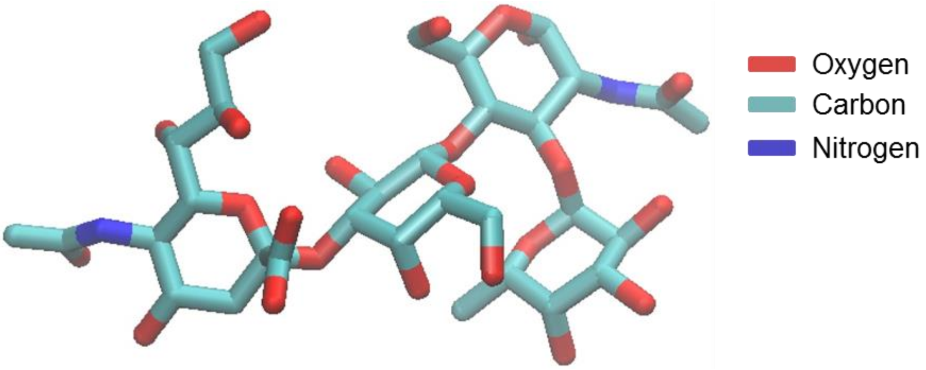

Figure S1. Schematic 3-D chemical structure of SLeX agent. For docking simulations and affinity calculations, the structure of SLeX was extracted from a protein complex defined in the Protein Data Bank (PDB) (PDB ID: 3PVD).

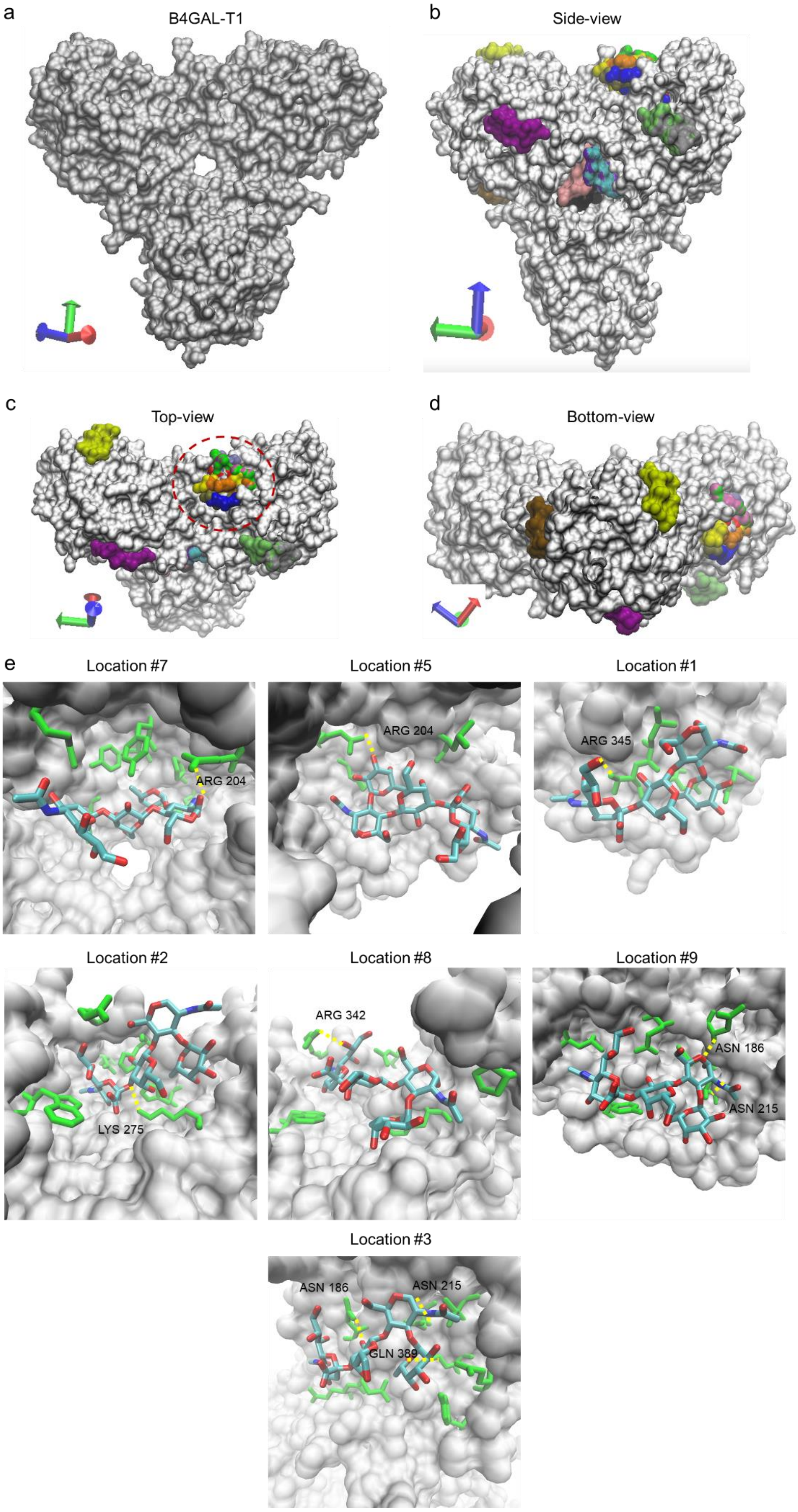

a
B4GAL-T1
b
Side-view
c
Top-view
d
Bottom-view
e
Location #7
ARG 204
Location #5
ARG 204
Location #1
ARG 345
Location #2
LYS 275
Location #8
ARG 342
Location #9
ASN 186
ASN 215
Location #3
ASN 186
ASN 215
GLN 389

Figure S2. 3-D visualization of β1–4 galactosyltransferase 1 (B4GAL-T1) and evaluation of SLeX binding to B4GAL-T1. (a) For the docking simulation, B4GALT1 was extracted from human M340H-beta-1,4-galactosyltransferase-1 (M340H-B4GAL-T1, PDB ID: 4EE3). (b) B4GAL-T1 and SLeX interactions were computed using AutoDock Vina. B4GALT1 is a peripheral membrane receptor that is located at the extracellular surface of the lipid bilayer. The simulation revealed at least nine unique locations for seventeen potential binding modes for SLeX binding. (c-d) The binding of SLeX agents to specific locations on B4GALT1 was illustrated with different views. SLeX agents were visualized with different colors for each binding mode. (e) Detailed representative images for different binding locations.

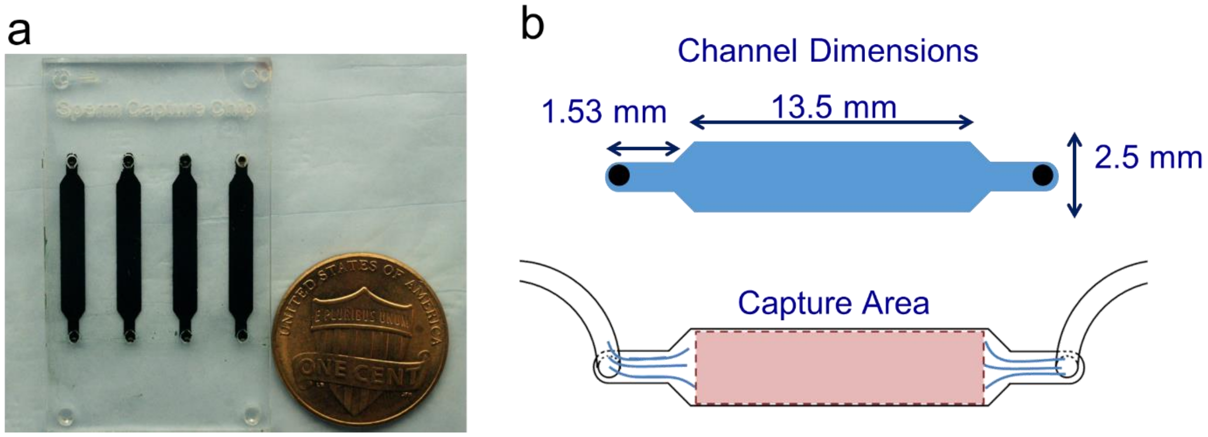

Figure S3. Microfluidic chip. (a) We designed a microfluidic chip with four channels. The chip has four inlets and outlets for sampling and washing steps. We introduced a food dye (dark blue-black) to visualize the channels. (b) The channel dimensions are demonstrated.

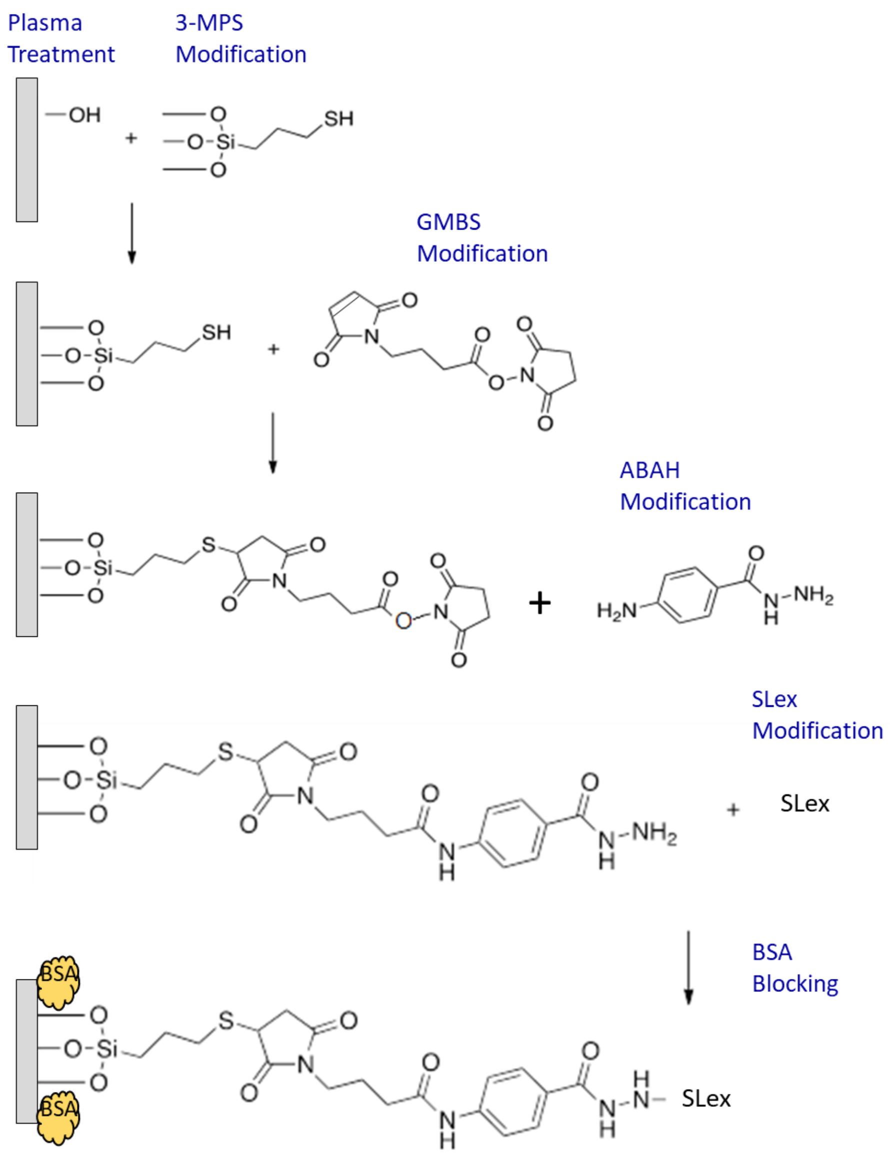

Figure S4. Surface chemistry. Surface chemistry is employed at the glass coverslip surface. Briefly, the coverslip surface is first treated with O_2 plasma to generate some radicals and oxygen groups for silanization with 3-mercaptopropyl-trimethoxysilane (3-MPS) molecule. Followed by the generation of thiol groups on the surface, N-(gammamaleimidobutyryloxy) succinimide ester (GMBS) and 4-Aminobenzoic hydrazide (ABAH) modifications are utilized for Sialyl-Lewis^{X} (SLeX) immobilization. To avoid and minimize non-specific binding, BSA is used as an anti-fouling (blocking) agent.

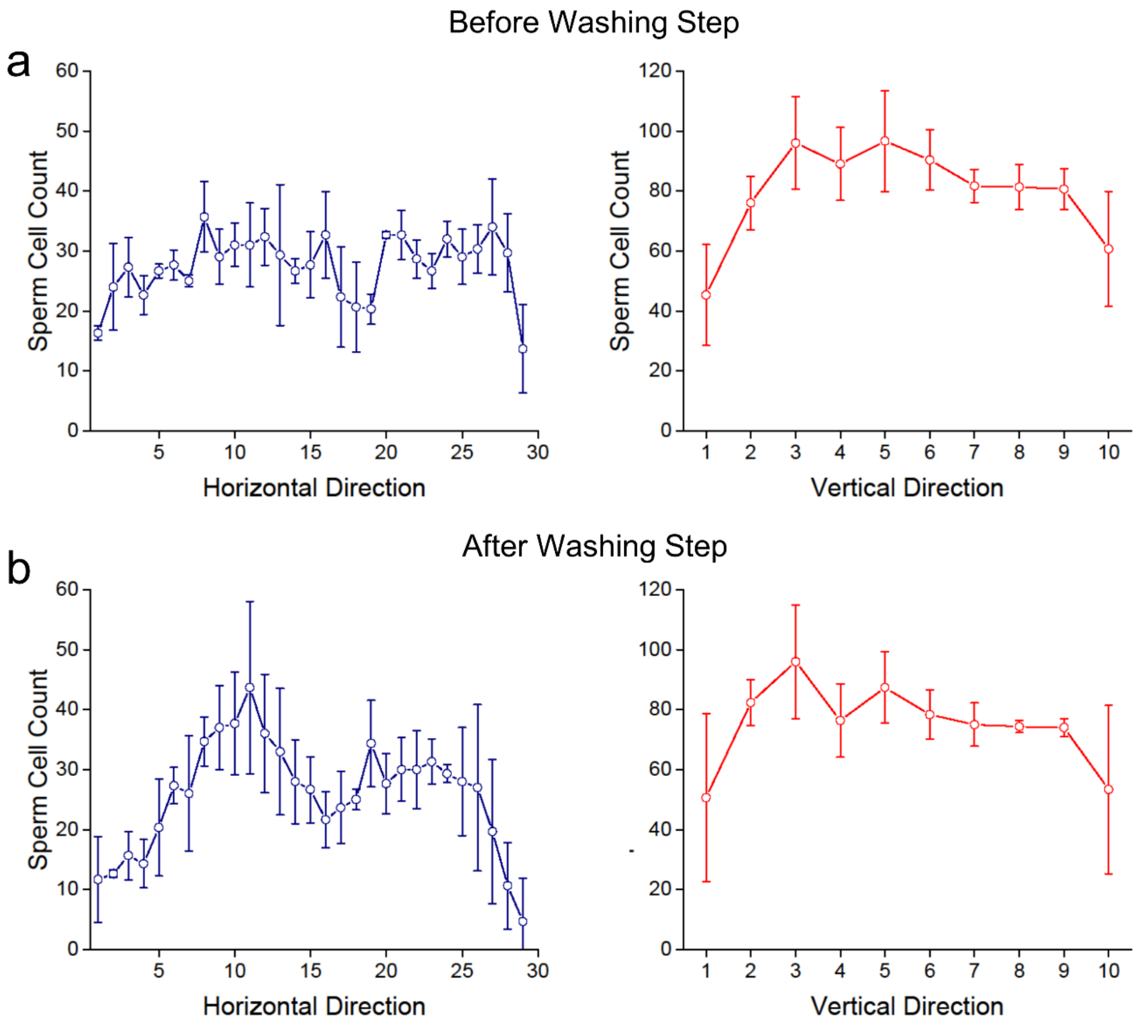

Figure S5. On-chip spatial distribution of low sperm count. (a-b) Sperm counts before and after washing steps were plotted in terms of horizontal and vertical directions. Before the washing step, nearly homogenous cell distribution was observed on the horizontal direction. Further, sperm cell count increased around the middle of the channels on the vertical axis. The cell count was slightly altered on the horizontal direction after the washing step. Most of the sperm close to the inlet released from the channel surface, while the distribution trend at the vertical axis remained constant after the washing step. Data was shown with average value ± standard deviation.

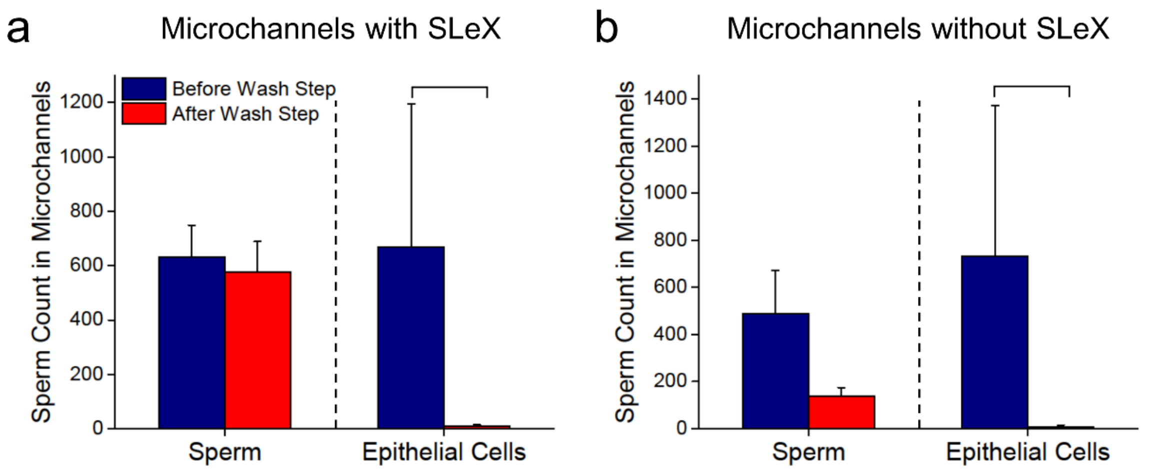

Figure S6. Evaluation of specificity parameter. We designed two sets of microfluidic chips: (i) the channels were modified with all surface chemistry steps including SLeX, and (ii) the channels were decorated without SLeX. We then applied a heterogenous cell population consisting of sperm and buccal epithelial cells into the channels. Before and after wash steps, we counted the cell numbers. (a) In experimental set (i), we counted 630 ± 119 sperm cell per channel and 575 ± 113 sperm remained in the channels after the washing step ($n=5$, $p>0.05$). We also noticed that a significant number of epithelial cells were removed after a single wash step, and only 10 ± 5 epithelial cells remained ($n=5$, $p<0.05$). (b) In experimental set (ii), we counted 487 ± 187 sperm cell per channel and 139 ± 37 sperm remained in the channels after the wash step ($n=5$, $p>0.05$). We also noticed that epithelial cells were significantly removed after a single wash step, and only 8 ± 7 epithelial cells remained in the channels ($n=5$, $p<0.05$). One-way ANOVA with Tukey's post hoc test was used in statistical assessments of equal variances for multiple comparisons. Horizontal brackets represented statistically significant differences between groups, and data was shown with average value ± standard deviation.

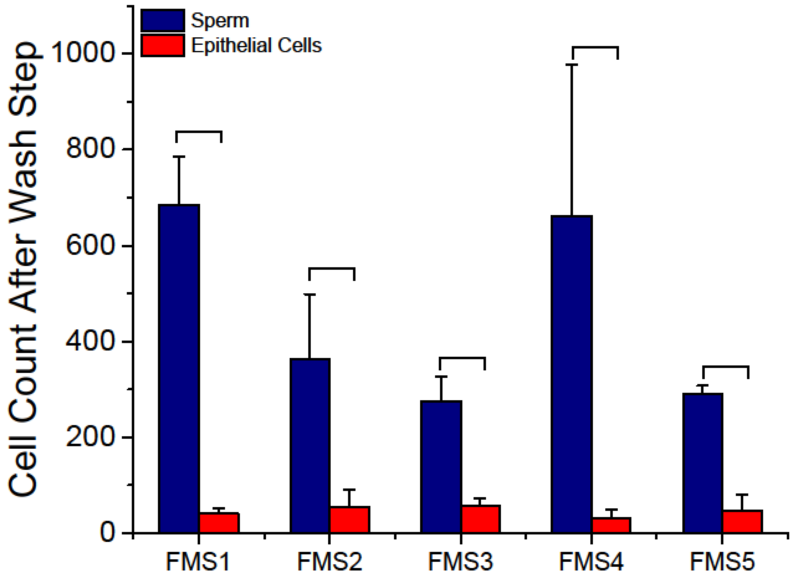

Figure S7. Validation of microfluidic chips with forensic mock samples. Simulated forensic samples (non-casework samples) were collected from Broward Sheriff's Office Forensic Laboratory. The microfluidic chips were validated with five different mock samples, and sperm and epithelial cells were then counted before and after the washing steps. We observed that a significantly lower number of epithelial cells remained in the channels compared to the captured sperm count (n=3, $p<0.05$). One-way ANOVA with Tukey's post hoc test was used in statistical assessments of equal variances for multiple comparisons. Horizontal brackets represented statistically significant differences between groups, and data was shown with average value ± standard deviation. FMS represents "Forensic Mock Sample".

Supplementary Videos

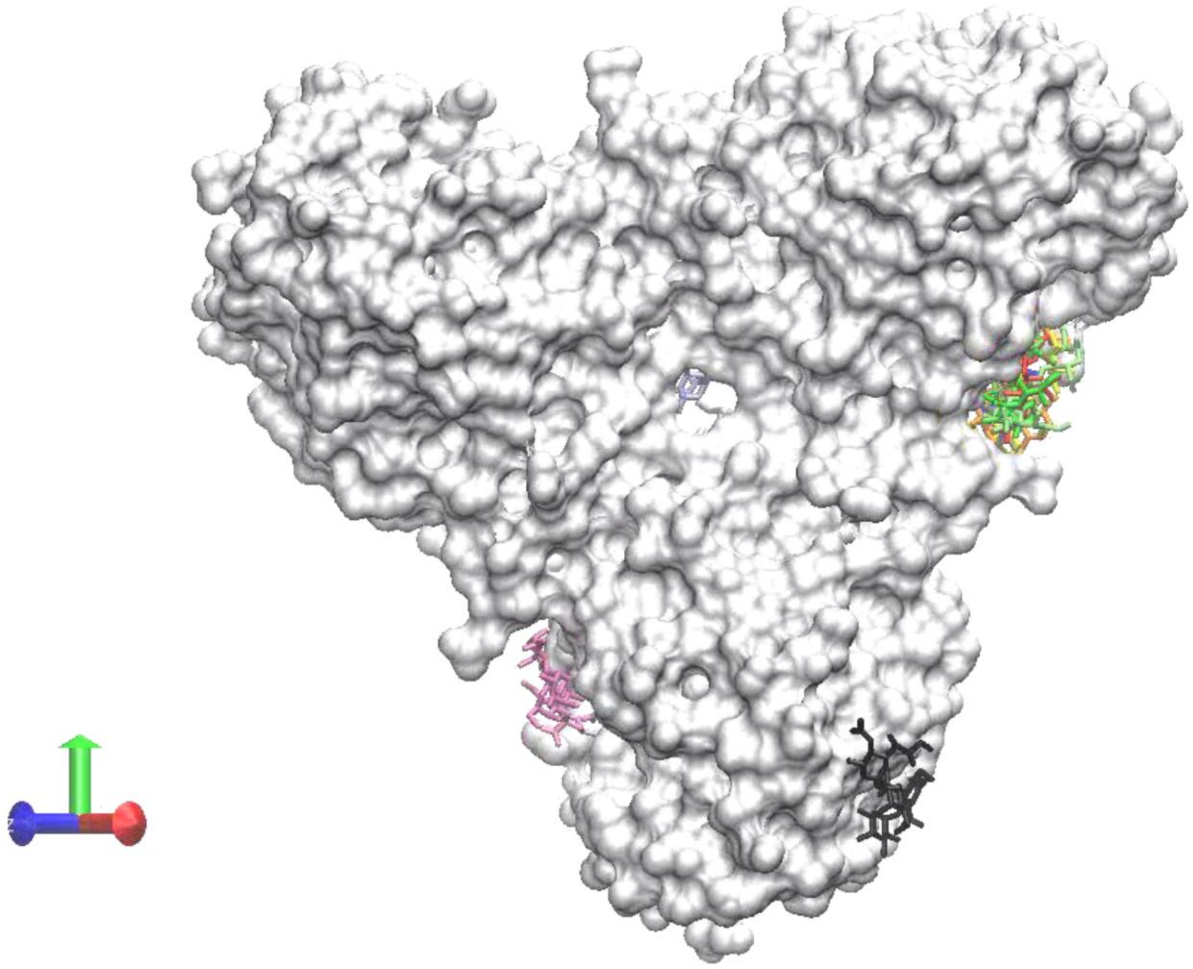

Video S1. Evaluation of SLeX binding kinetics and binding locations on sperm head. Various binding locations of SLeX were observed on β1–4 galactosyltransferase 1 (B4GALT1) located on sperm head.

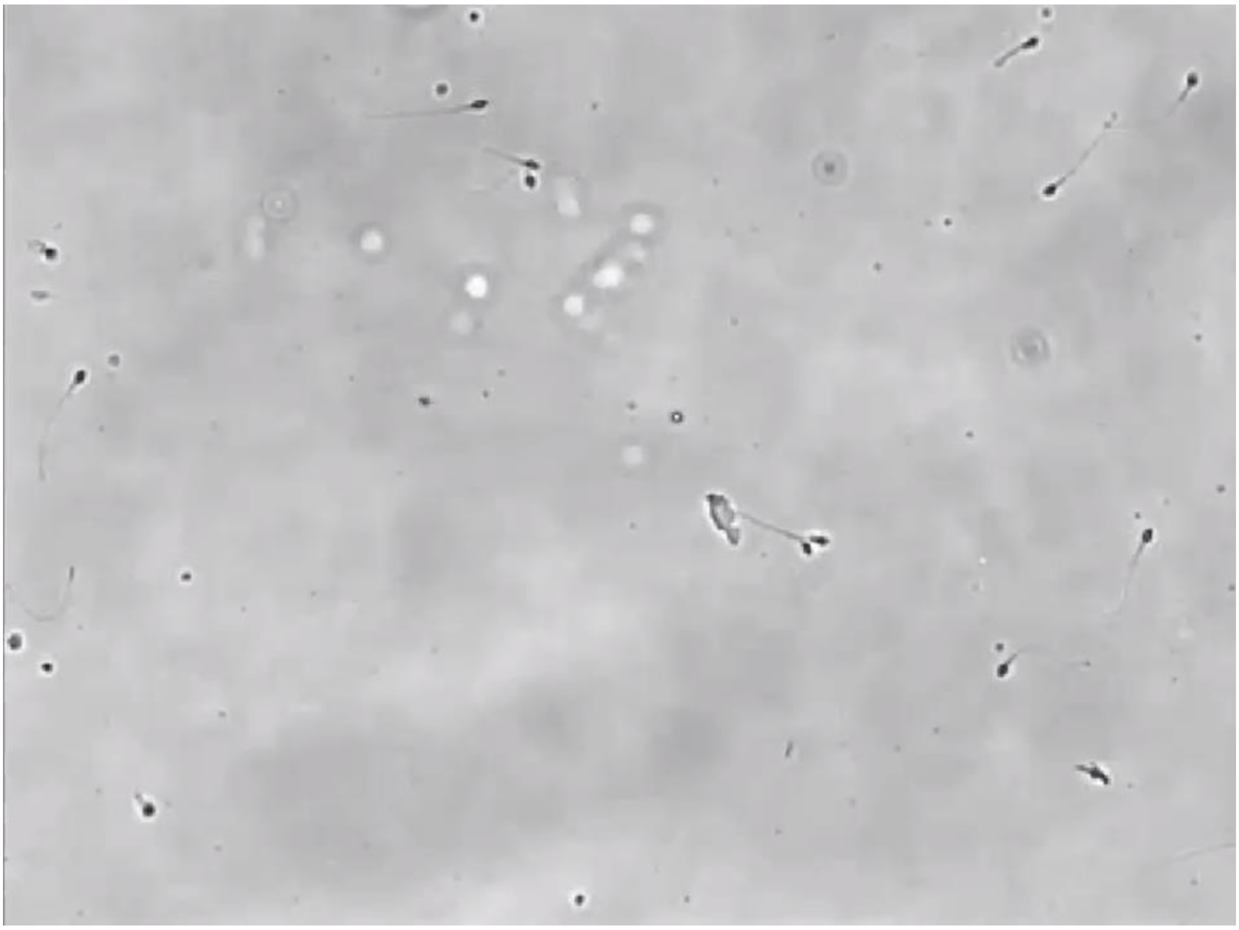

Video S2. Evaluation of sperm capture on SLeX-immobilized microchannels. After optimized surface chemistry was performed, sperm samples were applied into the microchannels and they were captured in the channels.
